# 非小细胞肺癌影像学特点与局部淋巴结转移之间的相关性

**DOI:** 10.3779/j.issn.1009-3419.2012.12.07

**Published:** 2012-12-20

**Affiliations:** 610041 成都，四川大学华西医院核医学科 Department of Nuclear Medicine, West China Hospital, Sichuan University, Chengdu 610041, China

**Keywords:** 肺肿瘤, 淋巴结转移, 危险因素, Lung neoplasms, Node metastasis, Risk factor

## Abstract

非小细胞肺癌（non-small cell lung cancer, NSCLC）局部淋巴结（N）分期是影响患者治疗方案选择的关键因素之一。目前临床所使用的无创和有创性N分期方法均有其局限性。研究发现NSCLC的某些影像学特点可预测淋巴结转移的危险性，包括大小、CT密度和氟代脱氧葡萄糖（fluorodeoxyglucose, FDG）标准化摄取值（standardized uptake value, SUV）等。期待系统性多因素分析，以发现影响肺癌淋巴结转移的关键因素。

## Abstract (en)

N staging is a key factor for the choice of treatment modalities in patients with non-small cell lung cancer. Both of the noninvasive and invasive N staging procedures have their own limitations. It is reported that some imageological characteristics have predictive value, including tumor size, CT attenuation and fluorodeoxyglucose (FDG) standardized uptake value (SUV), and so on. It is expected to find the key risk factors with multivariate analysis.

肺癌是目前最常见的恶性肿瘤，同时也是肿瘤死亡的首要病因。在我国，肺癌的死亡率仍处于上升趋势^[1]^。一般情况下，肺癌分为两种主要类型，即小细胞肺癌（small cell lung cancer, SCLC）和非小细胞肺癌（non-small cell lung cancer, NSCLC），其中NSCLC约占所有肺癌的80%^[2]^。局部淋巴结分期是影响NSCLC患者治疗方案选择的关键因素之一。对于N0-1期患者，手术是首选治疗方法；当术前发现N2淋巴结转移时，主要治疗方法则为放化疗或新辅助治疗+手术治疗^[2, 3]^。

目前临床使用最为广泛的无创性N分期方法是CT，主要依据淋巴结的大小来判断有无转移，其判断纵隔淋巴结转移的敏感性和特异性分别为51%和85%。正电子发射型计算机断层显像（positron emission tomography, PET）在纵隔淋巴结转移的诊断效能上较CT有了一定的提升，但总的敏感性也只有74%，而特异性与CT相同^[4]^。以往总是将PET表现为假阴性的原因归因于淋巴结的体积较小，但PET或PET/CT在诊断肿大淋巴结时的假阴性结果亦不罕见。*Meta*分析^[5]^发现，对于那些在CT上直径介于10 mm-15 mm而PET显示为阴性的淋巴结存在转移的概率为5%，而那些直径≥16 mm而PET显示为阴性的淋巴结存在转移的概率为21%。并且Lee等^[6]^发现，在CT和PET均显示为N0的NSCLC患者中，存在纵隔淋巴结转移的概率在T1期病变为6.5%，T2期病变为8.7%。研究^[7]^显示，通过测量淋巴结的表观弥散系数（apparent diffusion coefficient, ADC），MRI弥散加权成像（diffusion weighted imaging, DWI）评价NSCLC的N分期准确性高于PET/CT。但ADC值的评价需要获得较高的信噪比，发现小体积转移淋巴结的能力有限，目前通过计算ADC值来区分良恶性淋巴结病变尚处于研究阶段。

当无创性N分期方法不能满足需要时，临床工作者可求助于有创性分期方法，包括纵隔镜、经支气管穿刺活检、经支气管/食管超声引导下穿刺活检和胸腔镜等。上述方法的共同特点是适应症各有不同，即只能对纵隔内某些特定部位的淋巴结进行检查，存在各自检查的盲区。即使采用胸腔镜这种视野良好的检查方法，因为主动脉弓和肺动脉遮挡，在剥离左侧气管旁淋巴结时也较困难，故这些方法的敏感性为75%-90%，假阴性为7%-28%^[8]^。此外，上述方法可能会对患者造成不适或并发症，包括出血、感染、气胸、休克甚至死亡，多数检查需要全身麻醉，且对操作者个人技术及医院硬件要求均较高，不便于广泛开展。

鉴于目前临床所用的诸多N分期方法均存在不同的局限性，一些学者尝试发现一些可以在术前预测淋巴结转移可能性的危险因素，以期为NSCLC的临床分期和治疗提供更多有价值的参考信息。在影像学方面，目前研究较多的影响因素包括肺癌原发灶的大小、CT密度和FDG摄取值，本文就上述三种因素与转移之间的关系做简要总结。

## 1 肿瘤大小

在肺癌TNM分期系统中虽然肿瘤大小是影响T分期的一个重要因素，但该系统对患者临床分期的划分主要依据生存时间，并未考虑到肿瘤的大小与转移之间是否存在某种相关性。

为解决这个疑问学者们做了不少研究。Yang等^[9]^回顾性分析我国917例手术治疗的NSCLC患者发现，原发肿瘤越小，出现转移的可能性越小，当原发病灶直径≤20 mm、21 mm-30 mm、31 mm-50 mm、51 mm-70 mm及≥71 mm时，发生N1和N2淋巴结转移的几率分别约为25.3%、37.9%、49.7%、51.0%和61.5%。而Henschke等^[10]^对国际早期肺癌筛查项目（International Early Lung Cancer Action Program, I-ELCAP）中的436例NSCLC患者分析后发现，当原发灶≤15 mm、16 mm-25 mm、26 mm-35 mm及≥36 mm时，患N0M0期疾病的几率分别为91%、83%、68%和55%。由于该项目仅有2例患者出现了N3/M1转移，不难看出，原发肿瘤大小与肿瘤淋巴结转移情况之间存在强的相关性，原发肿瘤越大，其出现淋巴结转移的可能性就越高。有关肿瘤大小与转移之间相关性研究的最大样本是Wisnivesky等^[11]^对美国癌症研究所（National Cancer Institute, NCI）的监测、流行病学和最终结果注册表（Surveillance, Epidemiology and End Results Registry, SEER）数据库分析后获得的，该研究共分析了84, 152例NSCLC患者，结果显示，原发肿瘤越小，疾病临床分期越有可能是Ⅰ期，对于直径 < 15 mm、16 mm-25 mm、26 mm-35 mm、36 mm-45 mm和 > 45 mm的患者，Ⅰ期的百分比分别为54.1%、46.5%、34.4%、25.2%和14.7%，Ⅲ期的百分比分别为17.2%、21.1%、25.7%、30.6%、39.3%。因Ⅲ期NSCLC的主要构成是N2或N1患者，因此这一结果也可以理解为随原发肿瘤体积的增加，局部淋巴结转移的概率随之增加。

上述研究尽管方法和结果有所差异，但均发现NSCLC原发病灶大小与纵隔淋巴结转移存在正相关性，肿瘤越大，出现转移的可能性就越大。出现这种现象的原因可能是随着肿瘤体积增大，肿瘤血管和淋巴管生成增多，进入脉管内的肿瘤细胞也会增多，出现转移的几率也会增加。

## 2 CT密度

虽然NSCLC局部淋巴结转移与肿瘤的大小相关，但在所有T1期肿瘤中，仍有约20%存在淋巴结转移^[12]^。如能术前将那些在生物学上呈侵袭性的肿瘤和非侵袭性的肿瘤相鉴别，对行局限性肺切除术的患者极为重要。

薄层CT或高分辨CT（high-resolution CT, HRCT）在临床上的广泛应用极大地方便了对小肺癌病灶密度特点的观察，不少学者将目光转向肺癌的密度特点与转移之间关系的研究上。在HRCT上部分周围型小肺癌呈现为毛玻璃样密度（ground-glass opacity, GGO）。GGO在CT上表现为肺密度稍增高，但密度增高区域内的正常肺结构如支气管、血管和小叶间隔依然可以辨认。一般认为，GGO是肺原位腺癌（adenocarcinoma *in situ*, AIS）和微浸润腺癌（minimally invasive adenocarcinoma, MIA）的典型表现，其主要病理学特点是肿瘤细胞沿肺泡壁匍匐生长，侵袭性低，且预后较好^[13]^。混有GGO和实性成分的结节称为部分实性结节（part-solid）。研究^[14]^发现GGO在一个病灶内所占百分比与组织病理学所证实的低侵袭性成分相关。

多项报道^[14, 15]^显示在HRCT上GGO和实性成分所占比例与肿瘤的分期和预后呈强的相关性。早期肺癌筛查项目^[10]^显示，GGO、部分实性及实性NSCLC出现转移的几率分别为0、5%和21%。Aoki等^[14]^分析127例直径 < 30 mm的腺癌后发现，当病灶GGO比例分别占 > 50%、50%-10%和 < 10%时，其出现淋巴结转移的几率分别为4%、17%和26%。Matsuguma等^[16]^的结果也显示，GGO成分≥50%时，100%的肿瘤不伴淋巴结转移，而对GGO成分 < 20%的患者，35%存在淋巴管侵犯，32%伴发淋巴结转移。Nakata、Ikeda及Okada等^[15, 17, 18]^的研究也发现相似结果。

尽管GGO成分的比例可反映病灶的恶性程度，但需要指出的是在HRCT上对GGO特点的观察和量化主观性较强，而且观察者之间的不一致性可能会影响结果判断，并且在计算GGO所占比例时，不同的CT窗宽/窗位设置会直接影响量化结果。

与国外学者主要观察GGO在肺癌病灶内所占的比例不同，我国学者张等^[19]^对肺癌的绝对CT值（普通扫描）进行了研究，结果显示，相同病理学类型的肺癌CT值与淋巴结转移可能性之间存在负的相关性，CT值越低，越易发生淋巴结转移。增强CT扫描时，邓等^[20]^发现肺癌CT增强灌注强化指标灌注值（perfusion value, PV）与淋巴结转移之间也存在相关性，淋巴结转移组的PV高于无淋巴结转移组，且有统计学意义，当以PV > 1.52 mL/min/mL作为存在淋巴结转移的诊断指标时，敏感性为84.6%，特异性为85.2%，准确性为84.9%。但肺癌的CT增强PV通过何种机制对淋巴结转移产生影响有待进一步研究。

## 3 FDG摄取值（standardized uptake value, SUV）

肿瘤的大小和密度只能用来描述肿瘤的一些外源性特征，并未将肿瘤的内源性特点考虑在内。临床上肺癌常表现局部侵袭、局部淋巴结扩散、肺内转移和全身播散等四种生长方式，这也有可能代表了肺癌的四种生物学行为^[21]^。但在初诊时，除非已经发生转移，否则很难预测肿瘤以后会以哪种或哪几种生长方式在人体内继续存在。并且目前对有关发现小的“早期”肺癌是否会改善患者的预后尚存在争议。研究^[22]^发现，当肿瘤仅为1 mm-2 mm大小时，即会出现血管生成，进而使肿瘤细胞进入血液循环。一旦1个NSCLC病灶长到可被当代CT扫描仪所发现并做出诊断的尺寸，其大小也会远大于1 mm-2 mm，即使能发现并得到及时诊断，也可能是已经出现远处转移^[20]^。因此理想的分期系统应能反映肿瘤的生物学特性，在治疗前将患相同生物学特性的肿瘤的患者归类，并有针对性地进行治疗、随访，且这种分类方法应比现有的根据解剖学特征分类更实用、更有效。但是，目前人类预测肿瘤生物学行为的能力有限，对肿瘤的哪些生物学特征是真正的关键因素尚缺乏了解。作为一种代谢-分子显像方法，肿瘤在PET上表现出的FDG浓聚可能是内源性因素之一。

自^18^F-FDG PET应用于临床以来，其在评价肺结节、局部淋巴结转移、远处转移和疗效评价方面已成为了一种越来越有用的无创性影像学检查方法。常用参数SUV是目前间接反映肿瘤葡萄糖代谢水平的最常用的半定量指标。近期研究^[23]^发现，SUV对NSCLC患者预后有预测价值。此外，由于SUV的高低常标志着病变的代谢活性，不少学者认为如果SUV与转移可能性之间存在相关性，通过对NSCLC分期时兼顾原发灶的SUV，肺癌分期的准确性可能会得到提高，并且这种关联可能会成为FDG摄取水平有预后价值的基础。

Cerfolio等^[24]^对315例NSCLC患者进行多因素分析后发现，原发病灶的最大SUV（SUV_max_）与N分期呈正相关，并且是一个独立的预测因素，此外它也能独立地预测肿瘤脉管侵犯的可能性，而后者是肿瘤转移的关键步骤。Higashi等^[25]^的多中心临床研究显示，NSCLC的淋巴管侵犯和淋巴结转移取决于肿瘤摄取FDG的水平，并且推断原发灶摄取FDG是淋巴管侵犯和淋巴结转移的一个强有力的预测因子。Nambu等^[26]^研究66例NSCLC患者发现，淋巴结转移的可能性随着原发病灶的SUV_max_增加而增高，当原发病灶的SUV_max_ < 2.5时，发生淋巴结转移的几率为0，而当SUV_max_ > 12时，无论淋巴结FDG摄取水平有多高，其发生淋巴结转移的可能性可达70%。这个发现使淋巴结转移的预测更加敏感，其中包括那些不能通过直接分析每组淋巴结的影像学特点而发现的镜下转移。然而该项研究缺乏某一上限值，即当原发灶的SUV_max_高于此值时，总会出现淋巴结转移。因此，一个高的SUV_max_并不意味着总伴有淋巴结转移，另一方面，由于NSCLC原发灶SUV_max_ < 2.5时出现淋巴结转移的可能性不大，因此，当淋巴结摄取FDG很浓而原发灶SUV_max_ < 2.5时，应首先排除假阳性的可能。

由于GGO部分的肿瘤生长速度慢，并且单位体积内的肿瘤细胞密度较低等原因，常导致含有GGO成分的肿瘤SUV_max_偏低。但Lee等^[27]^对CT显示为部分实性的肺腺癌患者研究后发现，原发灶的SUV_max_依然是影响淋巴结转移的一个独立因素。此外，Okada等^[28]^对60例肺腺癌患者研究后发现，与GGO成分所占比例相比，SUV_max_与脉管侵犯和淋巴结转移的关系更紧密。这些结果提示，SUV_max_是肺腺癌侵袭能力的标志，而PET则可能是一个有力的检测工具，利用该方法可以识别哪类患者存在较高的淋巴结转移危险性。GGO成分的比例与低侵袭性肿瘤的生长密切相关，但肿瘤的SUV_max_与该部分的相关性较小。肿瘤干细胞生物学概念提示肿瘤最重要的部分是其最具侵袭性的部分，而这一部分可能并不占据整个肿瘤，也就是说SUV_max_并不反映病理学上的低侵袭性成分，而是反映最具侵袭性的那部分，因此，SUV_max_可能是肿瘤恶性程度高低的一个强有力的预测因子。

## 4 结语

除肿瘤的大小、CT密度和FDG摄取水平外，不少研究还发现，原发肿瘤的组织学类型等因素也与NSCLC的N分期相关，并且也有研究^[6, 29, 30]^发现肿瘤的SUV_max_与大小、密度、组织病理学特点之间存在相关性。因此，就很难断定究竟是哪一个因素影响了肿瘤的局部淋巴结转移，亦或均为独立的影响因素。临床上还可能存在影响肿瘤转移的其它因子，期待包括更多影响因素的系统性多因素分析，以发现影响NSCLC淋巴结转移的关键因素。
